# Clinical Outcomes of Very Short Term Dual Antiplatelet Therapy in Patients With or Without Diabetes Undergoing Second-Generation Drug-Eluting Stents: A Systematic Review and Meta-Analysis of Randomized Clinical Trials

**DOI:** 10.3389/fcvm.2021.655718

**Published:** 2021-07-01

**Authors:** Xi-Ying Liang, Yan Li, Xuan Qiao, Wen-Jiao Zhang, Zhi-Lu Wang

**Affiliations:** ^1^The First Clinical Medical College of Lanzhou University, Lanzhou, China; ^2^Department of Cardiology, The First Hospital of Lanzhou University, Lanzhou, China

**Keywords:** coronary artery disease, diabetes mellitus, dual antiplatelet therapy, drug eluting stents, percutaneous coronary intervention

## Abstract

**Background:** Patients with diabetes represent 20–30% of the population considered for percutaneous coronary intervention (PCI) and associate with more deleterious clinical outcome, which requires the optimal strategy of dual antiplatelet therapy (DAPT). The meta-analysis aims to compare clinical outcomes between very short (1–3 months) and standard (12 months) DAPT after implanting the second-generation drug-eluting stents in patients with or without diabetes following PCI.

**Methods and Analysis:** PubMed, Embase, Web of Science, Ovid, Cochrane Library, and ClinicalTrials.gov were searched for studies comparing the very short term and standard DAPT in patients with or without diabetes following PCI. Risk ratio with 95% confidence intervals was used to evaluate the pooled effect of discontinuous variables, and the pooled analyses were performed with RevMan 5.3 and Stata SE 14.0 software.

**Results:** A total of 38,864 patients were randomized to the very short term DAPT (*N* = 19,423) vs. standard DAPT (*N* = 19,441). Among them, 11,476 patients were diabetes and 27,388 patients were non-diabetes. The primary outcome of the net adverse clinical event (NACE) was significantly lower in diabetic patients with very short term DAPT (risk ratio 0.72, 95% CI 0.60–0.88, *p* = 0.0009). The same result was also found in the major cardiac or cerebrovascular events (MACCEs) (0.87, 0.78–0.98, *p* = 0.03). The risk of major or minor bleeding was significantly reduced in very short term DAPT regardless of the diabetes statue (0.69, 0.52–0.93, *p* = 0.01 in the diabetic group, and 0.50, 0.39–0.63, *p* <0.0001 in the non-diabetic group). There was no statistical difference in the incidence of major bleeding, all-cause death, cardiac death, myocardial infarction, definite or probable stent thrombosis, and stroke between the very short term DAPT (1–3 months) and standard DAPT (12 months) in patients with or without diabetes.

**Conclusion:** The very short term DAPT can significantly reduce the risk of the NACE and MACCE in patients with diabetes compared to standard DAPT. Meanwhile, the very short term DAPT can also reduce the incidence of major and minor bleeding without increasing the risk of ischemia in patients with or without diabetes (Registered by PROSPERO, CRD42020192133).

**Systematic Review Registration:** https://www.crd.york.ac.uk/prospero/, Identifier: CRD42020192133.

## Introduction

Diabetes mellitus is an independent risk factor for coronary artery disease, and its prevalence continues to rise worldwide (1), increasing the morbidity and mortality of coronary artery disease (2). The coronary artery lesions in patients with diabetes are usually diffuse and involve smaller-caliber vessels, which can influence the response to revascularization and lead to more harmful clinical outcomes (3–5). In the era of bare metal stents, diabetes mellitus is a strong predictor of adverse prognostic events, such as stent restenosis and unplanned revascularization (6). With the development of stent technology, the drug-eluting stent (DES) significantly reduced the incidence of target vessel revascularization and did not increase the risk of stent thrombosis in diabetic patients after percutaneous coronary intervention (PCI) (7). Particularly, the application of the second-generation DES further reduced the risk of very late stent thrombosis compared to the first-generation DES (8).

The first-generation DES with high thrombotic rate emphasized prolonging dual antiplatelet therapy (DAPT), especially in a high-risk population of ischemia (9, 10). The 2019 European Society of Cardiology (ESC) guideline on diabetes and cardiovascular diseases recommended that 1 year DAPT should be performed in patients undergoing PCI, and a prolongation of DAPT (beyond 12 months and up to 3 years) should be considered in diabetic patients who tolerated DAPT without major bleeding complications (1). However, several randomized controlled trials and meta-analysis have shown that compared with short-term DAPT, prolonging the duration of DAPT (>12 months) did not reduce the risk of ischemia but increased the risk of bleeding (11, 12). Over the past decades, due to the progress of stent technology and the update of antithrombotic therapy strategy, it is necessary to examine this issue from a new perspective. The current guidelines of the American College of Cardiology (ACC)/American Heart Association (AHA) and ESC recommend that the 1–3-month DAPT is only suitable for patients with stable coronary disease and high risk of bleeding (Class II), which was based on limited data (13, 14). Although there are some controversies on the duration of DAPT, the guidelines suggested that the strategy of DAPT should not be changed for patients with diabetes. However, the current recommendations lack data on very short term DAPT, especially in diabetic patients with a high risk of ischemia. Recent randomized controlled trials have enriched the landscape by investigating a very short term DAPT regimen, whereas the results are controversial (15–22).

In this background, the meta-analysis of randomized controlled trials was performed to assess the efficacy and safety of very short term (1–3 months) vs. 12-month DAPT in patients with and without diabetes undergoing DES implantation. The results suggest that the very short term DAPT can reduce the incidence of the net adverse clinical event (NACE) and major cardiac or cerebrovascular event (MACCE) outcomes in diabetes patients undergoing second-generation DES, and it reduces the risk of bleeding without increasing the risk of ischemia regardless of diabetes status.

## Methods

### Data Source and Quality Assessment

The present meta-analysis of randomized controlled trials was performed according to the Preferred Reporting Items for Systematic reviews and Meta-Analyses (PRISMA) statement (23). PubMed, EMBASE, Web of Science, Ovid, Cochrane Library, and ClinicalTrials.gov were searched for studies comparing the very short term with standard DAPT in diabetes or non-diabetes patients undergoing second-generation DES, without language restrictions from inception to June 19, 2020. Keep up with the latest publications by creating update alerts in the databases. The Medical Subject Headings (MeSH) terms of “coronary artery disease,” “drug eluting stents,” “percutaneous coronary intervention,” “diabetic mellitus,” “dual antiplatelet therapy,” and “randomized controlled trial” and other text words of “DAPT,” “dual anti-platelet therapy,” “DES,” “PCI,” “coronary artery disease,” “RCT,” and “diabetes mellitus” were searched. The inclusion criteria were as follows: (a) randomized controlled trials; (b) compared very short term (1–3 months) and standard (12 months) DAPT in patients with or without diabetes undergoing second-generation DES; (c) reported adverse outcomes of diabetes mellitus and non-diabetes mellitus separately as their clinical endpoints; (d) included studies were followed for at least 1 year. The exclusion criteria included the following: (a) meta-analysis, case studies, or editorials; (b) did not report the status of diabetic subjects in the population included; (c) the adverse outcomes of diabetes mellitus and non-diabetes mellitus were not reported as their clinical endpoints, respectively; (d) compared 6 months vs. 12 months or 12 months vs. prolonged DAPT. The title, abstract, and full text were independently screened by two investigators (LX-Y and LY) according to the inclusion and exclusion criteria mentioned above. Discrepancies were resolved through negotiations with a third party (WZ-L). The general quality of each study included was assessed according to the Cochrane tool of Collaboration for assessing the risk of bias (24). The Grading of Recommendations, Assessment, Development, and Evaluation (GRADE) approach was used to assess the quality of each endpoint (25). The study protocol was registered in PROSPERO (CRD42020192133). As analyses were based on previously published studies, ethical approval and patient consent are not required.

### Data Acquisition and Clinical Endpoints

The baseline characteristics of patients and trials as well as outcome data for each eligible study were extracted without modification by two investigators (LX-Y and LY) independently. The discrepancies were resolved through discussion. The primary outcome was 1 year of NACE, defined as a composite of bleeding and adverse cardiac and cerebrovascular events (specific definitions were based on the original study). The secondary outcomes were the bleeding and other outcomes. The bleeding outcomes included major bleeding and major or minor bleeding; the definitions were based on the original studies. Other secondary outcomes included MACCE, all-cause death, cardiac death, myocardial infarction, definite or probable stent thrombosis, and stroke within 1 year. The myocardial infarction was defined by the original trial. Stent thrombosis was defined by the Academic Research Consortium (26). Second-generation DES was described according to the original article. The outcome definition in each trial is reported (Supplementary Table 1).

### Statistical Analysis

The risk ratio and 95% confidence interval of each outcome were calculated for the pooled analysis. The continuous variables were reported as means or medians, and categorical variables were reported as percentages. The heterogeneity was assessed using Cochrane Q statistic with Pearson chi-square test and the Higgins *I*^2^ test. Random-effects model was performed to calculate the pooled risk ratio. According to the diabetes management strategy (whether insulin dependent or not), clinical manifestation (whether acute coronary syndrome or not), antiplatelet therapy after very short term DAPT (P2Y12 or aspirin), and different P2Y_12_ in DAPT (ticagrelor or clopidogrel), subgroup analysis was performed to explore their effect on clinical outcomes. In addition, the sensitivity analysis was employed to detect the impact of any single study result on the overall results. Two-tailed *p*-values were exploited, and *p* <0.05 was considered significant for all analyses. The meta-analysis was performed by Review Manager Version 5.3 software (The Nordic Cochrane Center, Copenhagen, Denmark) and Stata SE 14.0 (StataCorp LP, College Station, Texas). Visual estimation of funnel plot and the Begg's and Egger's tests were implemented to investigate the possibility of publication bias.

## Results

### Search Results and Characteristics

**Figure 1:**
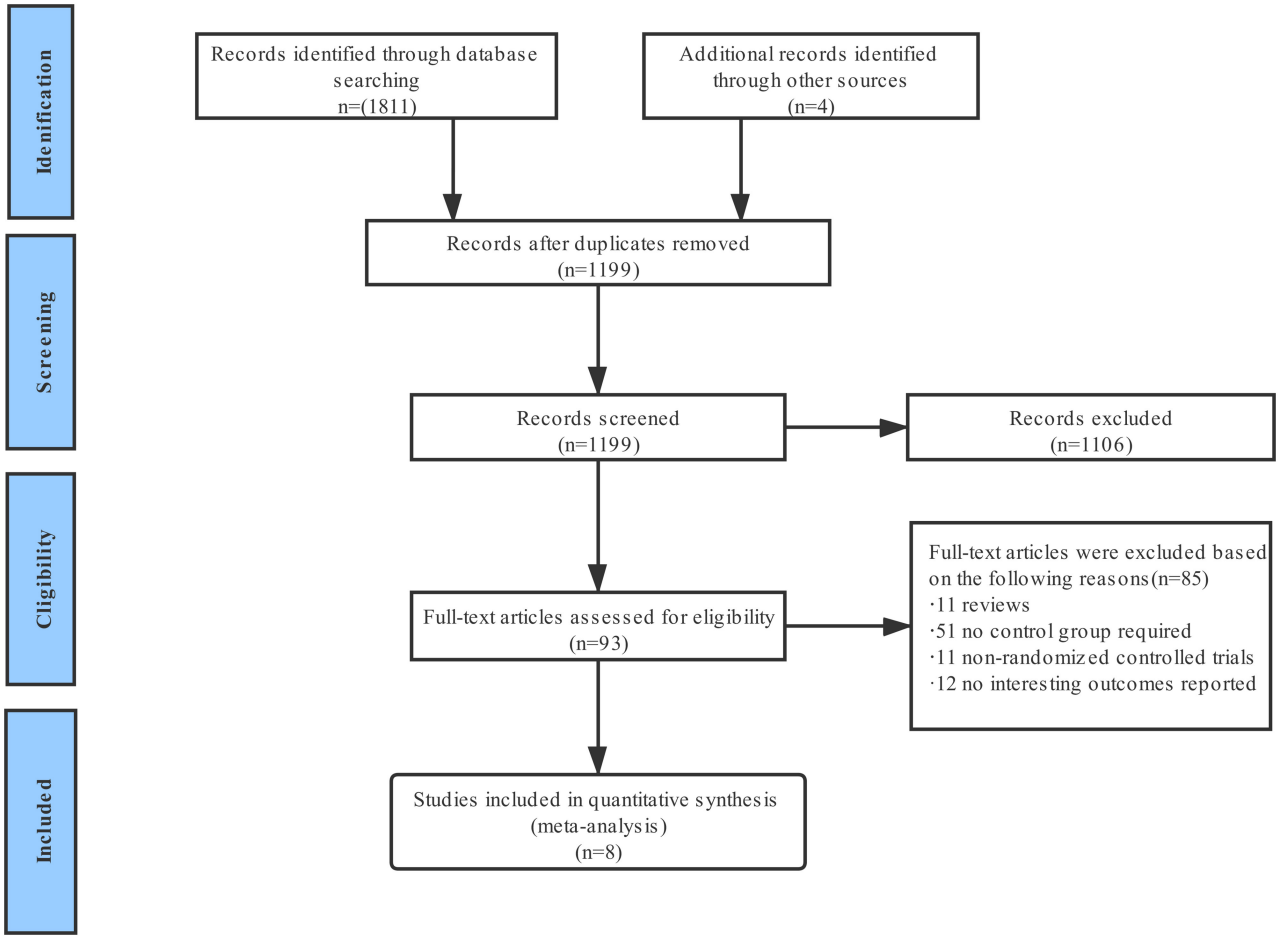
Flow diagram of literature searching process.

A total of 1,815 publications were found during the initial search, and 93 articles were further read full text after being initially identified by screening the title and abstract; finally, eight randomized controlled trials are included for final analysis (Figure 1) (15–22). The inclusion and exclusion criteria as well as primary outcomes of the studies included are summarized (Supplementary Table 2). These studies on diabetes mellitus were all in the subgroup of randomized clinical trials. Two of them were a pre-specified subgroup of diabetes mellitus, and three trials were stratified by the presence of diabetes mellitus. Among them, two trials compared 1 month with 12 months of DAPT, whereas six trials compared 3 months with 12 months of DAPT. Three trials used P2Y_12_ inhibitor monotherapy after DAPT, whereas five trials used aspirin monotherapy after DAPT. The median follow-up period was 12 months. The baseline characteristics of the trials included are shown (Table 1). A total of 38,864 patients were divided into the very short term DAPT group (*n* = 19,423) vs. standard DAPT group (*n* = 19,441). In addition, 11,476 patients were diabetes mellitus and 27,388 patients were non-diabetes mellitus. The utilization rate of second-generation DES ranged from 85% to 100%. The clinical and other baseline characters are summarized (Table 2). The average age of the patients included was between 60.5 and 68.6 years, and more than half of them were male. The patients with acute coronary syndrome varied from 37.3 to 100%. The incidence of multivessel cardiovascular disease was 33.8–61.6%, and the incidence of multivessel intervention was 6.7–26.5%. After treatment, each patient had an average of 1.1–1.5 lesions, and 1.2–1.6 stents were implanted. The lengths of stents ranged from 23 to 40.1 mm. The baseline characteristics for patients with or without diabetes are summarized (Supplementary Table 3), including only two trials. The results indicated that compared with patients with non-diabetes, patients with diabetes were older and were associated with higher body mass index and cardiovascular risk factors, such as hypertension, hyperlipidemia, and previous cardiovascular events. There was no significant difference in the number of lesions treated, multivessel PCI, and total stent length between the two groups. Compared with patients with non-diabetes, the average stent diameters per patient and stent number implanted per patient were higher in patients with diabetes.

**Table 1:** Characteristics of randomized controlled trials included.

**Study**	**TICO**	**TWILIGHT**	**STOPDAPT-2**	**SMART-CHOICE**	**REDUCE**	**GLOBAL LEADERS**	**OPTIMIZE**	**RESET**
**Year**	**2020**	**2020**	**2019**	**2019**	**2019**	**2018**	**2013**	**2012**
Setting	South Korea	11 countries	Japan	Korea	Europe and Asia	18 countries	Brazil	Korea
Multicenter	Y	Y	Y	Y	Y	Y	Y	Y
Blind method	open label	double-blind	open label	open label	open label	open label	open label	open label
Time to randomization	At index PCI	3 m after PCI	1 m after PCI	3 m after PCI	Before PCI	At index PCI	At index PCI	At index PCI
Prespecified diabetes subgroup	—	Y	—	—	—	Y	—	—
Stratified by diabetes	Y	—	—	—	—	—	Y	Y
Follow-up time (m)	12	15	12	12	12	24	12	12
Strategy of antiplatelet therapy	ASA+Tic 3 m + Tic 9 m vs. ASA+Tic 12 m	ASA+Tic 3 m + Tic 12 m vs. ASA+Tic 15 m	ASA+Clo 1 m + Clo 11 m vs. ASA+Clo 12 m	DAPT 3 m + P2Y^*^_12_ 9 m vs. DAPT 12 m	DAPT 3 m + ASA 9 m vs. DAPT 12 m	ASA+Tic 1 m + Tic 23 m vs. ASA+Tic 12 m + ASA12 m	ASA+Clo 3 m + ASA 9 m vs. ASA+Clo 12 m	DAPT 3 m+ASA 9 m vs. DAPT 12 m
Second generation stent (%)	100	98	100	99.9	100	100	100	85

*DAPT, dual antiplatelet therapy; PCI, percutaneous coronary intervention; ASA, aspirin; Tic, ticagrelor; Clo, clopidogrel; m, month(s)*.

* *P2Y12, ticagrelor, clopidogrel, or pragrelor. DAPT in the table was aspirin combined with ticagrelor, clopidogrel, or pragrelor*.

**Table 2:** Clinical, angiographic, and procedure characteristics of the included trials.

**Study**	**GLOBEL LEADERS**		**TWILIGHT**		**REDUCE**		**OPTIMIZE**		**RESET**		**TICO**		**STOPDAPT-2**		**SMART-CHOICE**	
	**1 m**	**12 m**	**3 m**	**15 m**	**3 m**	**12 m**	**3 m**	**12 m**	**3 m**	**12 m**	**3 m**	**12 m**	**1 m**	**12 m**	**3 m**	**12 m**
Number	7,980	7,988	3,555	3,664	751	745	1,563	1,556	1,059	1,058	1,527	1,529	1,500	1,509	1,495	1,498
Age, years (mean ± SD)	64.5 ± 10.3	64.6 ± 10.3	65.2 ± 10.3	65.1 ± 10.4	61	60	61.3 ± 10.4	61.9 ± 10.6	62.4 ± 9.4	62.4 ± 9.8	61 ± 11	61 ± 11	68.1 ± 10.9	69.1 ± 10.4	64.6 ± 10.7	64.4 ± 10.7
Female	23.4	23.1	23.8	23.9	17.4	22.7	36.5	36.9	35.6	37.1	21	20	21.1	23.5	27.3	25.8
BMI, kg/m^2^ (mean ± SD)	28.2 ± 4.6	28.2 ± 4.6	28.6 ± 5.5	28.5 ± 5.6	26.6	26.6	—	—	25.0 ± 3.2	24.9 ± 3.1	24.9 ± 3.2	24.9 ± 3.3	24.4 ± 3.5	24.2 ± 3.5	24.5 ± 3.1	24.7 ± 3.2
Hypertension	74	73.3	72.6	72.2	50.7	50.7	86.4	88.2	62.3	61.4	50	51	73.7	74	61.6	61.3
Hyperlipidemia	69.3	70	60.7	60.2	46.3	44.9	63.2	63.7	57.7	59.9	61	60	74.4	74.8	45.1	45.5
DM	25.7	24.9	37.1	36.5	21.6	19.5	35.4	35.3	29.8	28.8	27	27	39	38	38.2	36.8
Insulin-dependent	7.6	7.7	9.4	10.5	—	—	10.2	10.4	—	—	—	—	6.9	6.5	—	—
CKD	13.9	13.5	16.8	16.7	—	—	7.4	5.8	—	—	19	22	5.5	5.6	2.9	3.5
Smoking	25.9	26.3	20.4	23.1	42.1	42.7	18.6	17.3	25.2	22.8	36	38	26.6	20.6	28.4	24.5
Heart failure	—	—	—	—	—	—	4.3	4.2	11.3	11.8	—	—	7.7	7.1	—	—
PAD	6	6.7	6.9	6.8	—	—	2.8	3	—	—	—	—	6.4	6.6	—	—
Pre stroke	2.6	2.6	—	—	—	—	—	—	—	—	4	4	5.4	7	6.6	6.8
Pre MI	23	23.6	28.7	28.6	12.5	11.8	34.6	34.8	1.8	1.6	4	3	13.8	13.2	4.1	4.3
Pre PCI	32.7	32.7	42.3	42	11.7	9.8	20.9	19.1	3.5	3	9	8	33.5	35.1	11.5	11.8
Pre CABG	5.6	6.2	10.2	9.8	2.8	2.8	7.1	8.2	0.2	0.6	1	1	1.1	2.8	—	—
Anemia	—	—	19.8	19.1	—	—	—	—	—	—	—	—	8.1	9.4	—	—
Pre bleeding	0.6	0.7	0.9	0.9	—	—	0.6	0.6	—	—	—	—	1.3	1.9	—	—
SCAD	53	53.2	36.1	34.7	0	0	68.4	77.7	44.5	46.3	0	0	62.3	61.4	41.8	41.7
ACSE	47	46.8	63.9	65.7	100	100	31.6	32.3	55.5	53.7	100	100	37.7	38.6	58.2	58.3
UA	12.6	12.7	35.1	34.9	15.2	13.8	16.83	17.1	40.8	39.9	29	32	12.9	14.2	31.2	32.8
NSTEMI	21.1	21.1	28.8	30.8	35.6	41	5.4	5.4	14.7	13.8	35	32	5.4	6.6	16	15.4
STEMI	13.3	12.9	0	0	49.3	45.2	9.37	9.8	0	0	36	36	19.4	17.9	11	10
Radinal	73.9	74.2	73.1	72.6	76.1	76.9	—	—	—	—	55	56	82.1	83.8	73.0	72.8
Multivessel CAD	—	—	63.9	61.6	36.2	33.8	—	—	43.1	42.9	55	56	—	—	50.1	49
Multivessel intervention	25.5	25.3	—	—	17.4	18.1	25.34	26.54	22.0	23.4	17	18	6.7	7.7	22.5	24.6
**Location of lesions**																
Left Main	1.9	1.8	4.7	5.2	—	—	1.2	1.5	—	—	3	2	2.9	2.5	1.2	1.9
LAD	41.2	42	56.1	56.4	48.0	44.2	47.9	46.6	52.7	53.6	48	48	55.2	56.6	48.8	50.4
LCX	24.3	24.5	32.4	32.2	19.5	22.0	23.4	24.3	21	19.2	19	19	17.9	20.2	21.6	19.9
RCA	31.6	30.7	35	35.3	31.2	33.0	27.6	27.7	26.3	27.1	30	31	29.1	27.2	28.3	27.8
**Lesion complexity**																
Calcification	—	—	14	13.7	—	—	—	—	—	—	—	—	—	—	15.7	15.3
Bifurcation	12	12.1	12.2	12.1	—	—	14.7	14.9	—	—	14	15	25.1	26	13.3	12.1
Total occlusion	—	—	6.2	6.3	—	—	4.2	4.2	—	—	—	—	3.7	4.4	—	—
Thrombotic	4.6	5.3	10.4	10.7	12.5	13.6	—	—	—	—	4	5	—	—	7.4	7.5
Lesions treated per person	1.32	1.32	1.5 ± 0.7	1.5 ± 0.7	—	—	1.3 ± 0.6	1.3 ± 0.6	1.27 ± 0.53	1.27 ± 0.68	1.23 ± 0.50	1.24 ± 0.51	1.1 ± 0.4	1.1 ± 0.4	1.24	1.26
Stents treated per lesion	1.2 ± 0.5	1.2 ± 0.5	—	—	—	—	1.2 ± 0.5	1.2 ± 0.5	—	—	—	—	—	—	1.19	1.16
Stents treated per patients	—	—	—	—	1.20	1.21	1.6 ± 0.8	1.6 ± 0.8	—	—	1.37 ± 0.67	1.37 ± 0.67	1.3 ± 0.5	1.3 ± 0.6	1.47	1.47
Stent length, mm	24.8 ± 13.9	24.8 ± 14.0	40.1 ± 24.2	39.7 ± 24.3	23	23	32.75 ± 19.8	32.73 ± 20.0	19.6 ± 10.1	20.1 ± 10.8	35 ± 20	35 ± 21	30.3 ± 16.7	30.5 ± 16.8	38.0 ± 22.5	37.8 ± 22.9

*BMI, body mass index; DM, diabetes mellitus; PAD, peripheral arterial disease; CKD, chronic kidney disease; MI, myocardial infarction; PCI, percutaneous coronary intervention; CABG, coronary artery bypass grafting; SCAD, stable coronary artery disease; ACS, acute coronary syndrome; UA, unstable angina; STEMI, ST-segment elevation myocardial infarction; NSTEMI, non-ST-segment elevation myocardial infarction; LAD, left anterior descending; LCX, left circumflex artery; RCA, right coronary artery. Values shown are % (n) unless otherwise indicated*.

### Quality Assessment

The quality assessments of each randomized controlled trial included are presented (Supplementary Figure 1). All trials included were performed with the intention-to-treat analysis. Since most trials were open-label, a serious risk of bias was assembled. No other factors were found to reduce the quality of the study. The assessment of each outcome evidence quality with GRADE is shown (Supplementary Table 4). The evidence quality of all outcomes was moderate.

### The Primary Outcomes

**Figure 2:**
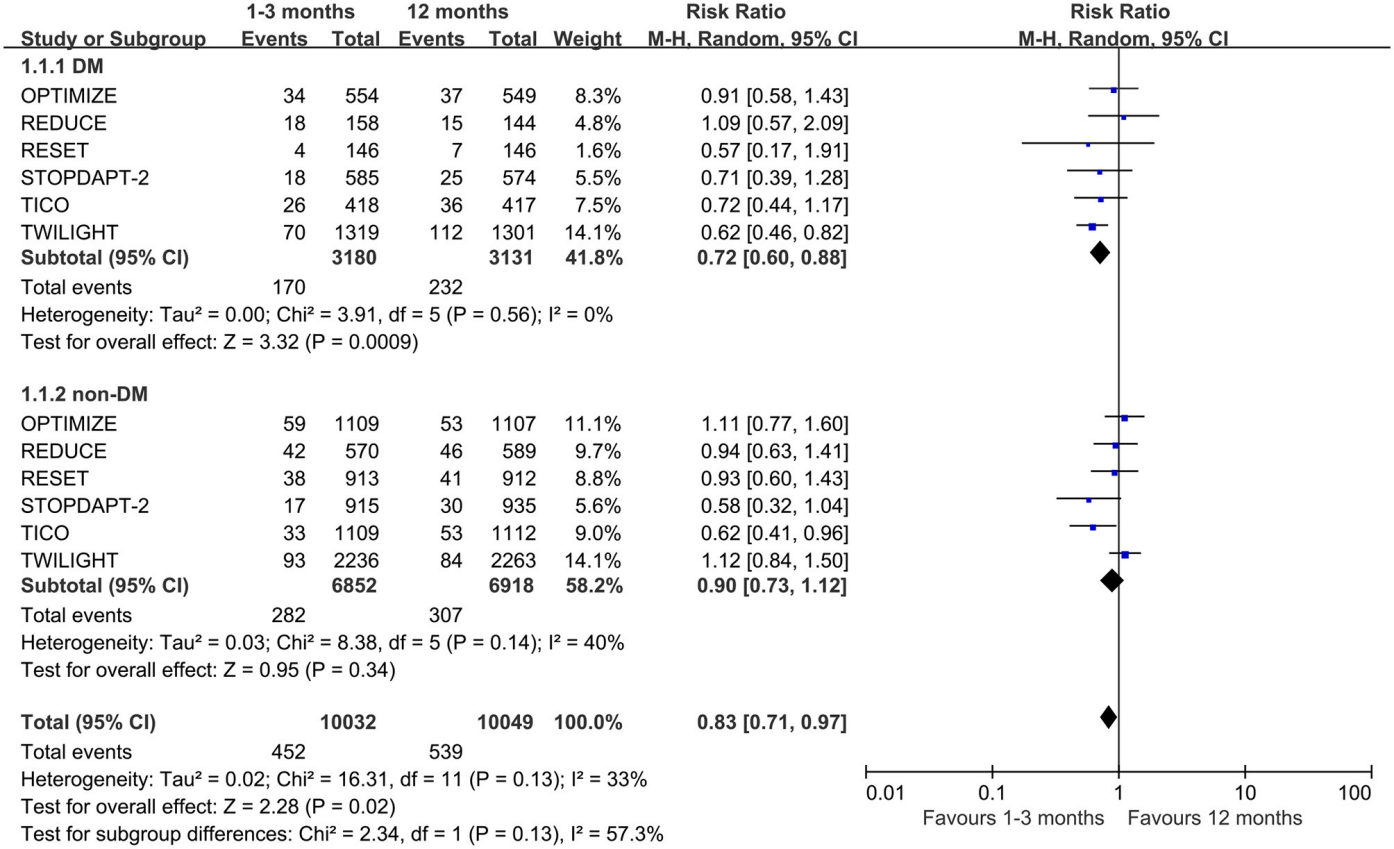
Forest plot of primary outcome (NACE) in patients with or without diabetes mellitus according to duration of DAPT.

Six of the eight trials reported the NACEs, involving 20,081 patients (6,311 with diabetes mellitus and 13,770 with non-diabetes mellitus). The pooled analysis demonstrates that the NACE of very short term (1–3 months) DAPT group was significantly lower than that of standard (12 months) DAPT group in patients with diabetes (0.72, 0.60–0.88, *p* = 0.0009, *I*^2^ = 0%), while there was no significant difference in patients with non-diabetes (0.90, 0.73–1.12, *p* = 0.34, *I*^2^ = 40%) (Figure 2). For diabetic patients, slight asymmetry is observed in the funnel plot (Supplementary Figure 2). For this result, the Begg's and Egger's tests did not show potential publication bias (*p* = 1.00 and *p* = 0.43, respectively). However, for non-diabetic patients, the results of two bias tests were different (*p* = 0.06 and *p* = 0.04 for Begg's and Egger's tests, respectively). Moreover, there was no significant interaction between the two groups of the NACE outcome (*p*_interaction_ = 0.13).

### Secondary Outcomes

#### Bleeding Outcomes

**Figure 3:**
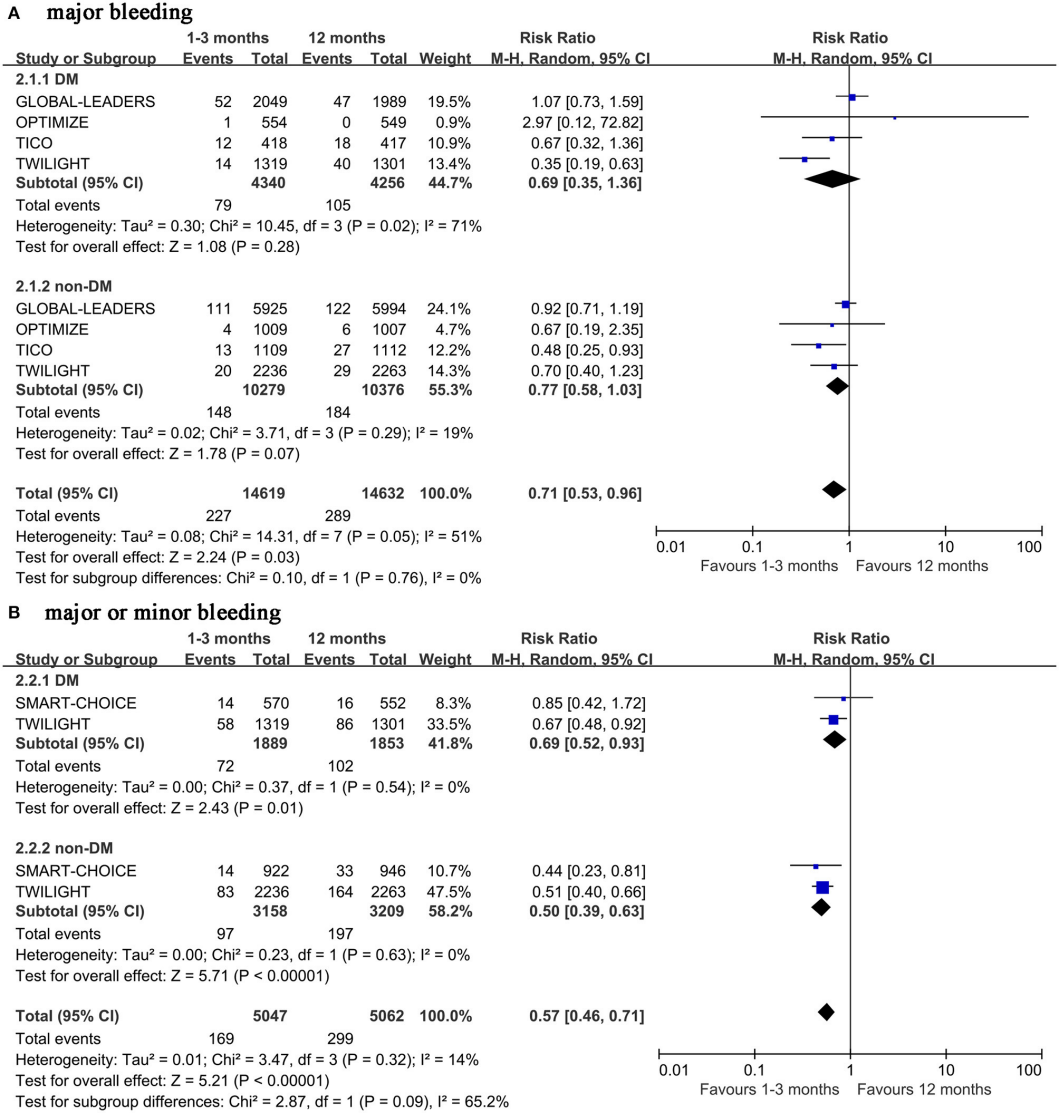
Forest plot of bleeding outcomes in patients with or without diabetes mellitus according to duration of DAPT.

The very short term DAPT does not reduce the incidence of major bleeding in patients with diabetes (0.69, 0.35–1.36, *p* = 0.28, *I*^2^ = 71%) (Figure 3A). The Begg's and Egger's tests did not show potential publication bias among the studies included (*p* = 1.00 and 0.89, respectively). Because of the high heterogeneity of the outcome, the sensitivity analysis was performed, which shows that the heterogeneity was reduced to 0% when the TWILIGHT study was removed, but the statistics did not change (0.98, 0.69–1.37, *p* = 0.89, *I*^2^ = 0%) (Supplementary Figure 3). The same results were also found in patients with non-diabetes (0.77, 0.58–1.03, *p* = 0.07, *I*^2^ = 19%). However, the very short term DAPT can reduce the incidence of major or minor bleeding outcomes regardless of diabetes mellitus status (0.69, 0.52–0.93, *p* = 0.01, *I*^2^ = 0% with diabetes mellitus, and 0.50, 0.39–0.63, *p* <0.00001, *I*^2^ = 14% with non-diabetes mellitus) (Figure 3B).

#### Other Secondary Outcomes

**Figure 4:**
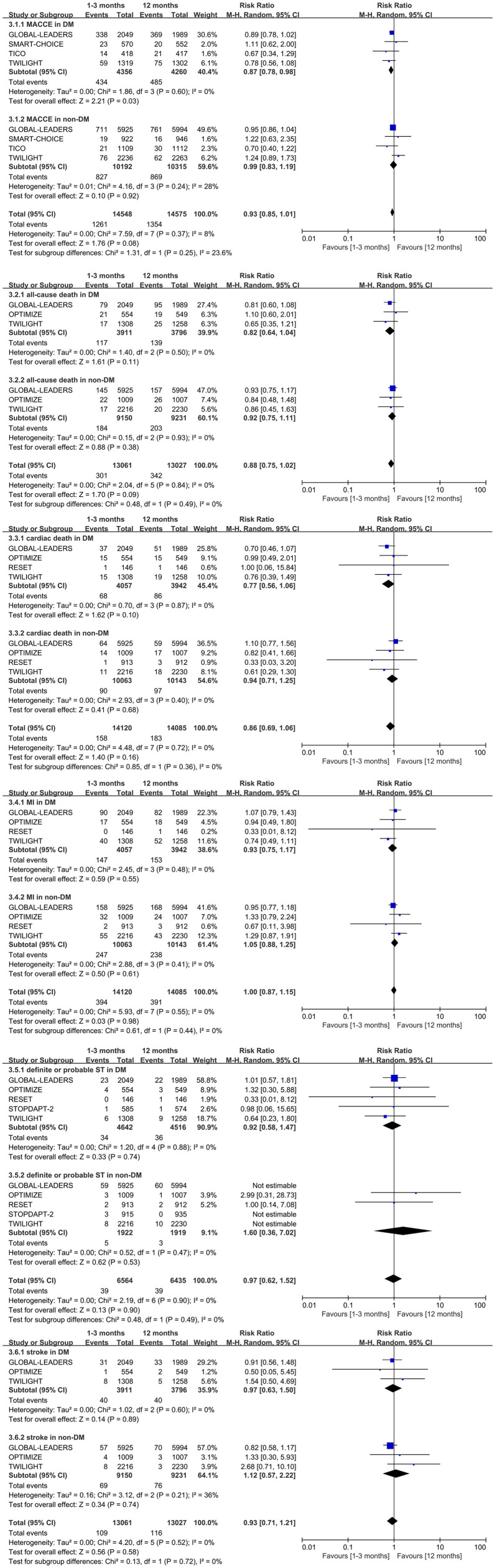
Forest plot of other secondary outcomes in patients with or without diabetes mellitus according to duration of DAPT.

The MACCE outcome was similar to the NACE. The very short term DAPT reduced the incidence of the MACCE by 13% in patients with diabetes (0.87, 0.78–0.98, *p* = 0.03, *I*^2^ = 0%). There is no significant difference between the very short term DAPT and standard DAPT groups in patients with non-diabetes (0.99, 0.83–1.19, *p* = 0.92, *I*^2^ = 28%) (Figure 4). Meanwhile, neither the Begg's nor Egger's tests found significant publication bias (*p* = 0.734, 0.724 in diabetes subgroup and *p* = 1.00, 0.718 in non-diabetes subgroup, respectively).

There is no significant difference in all-cause death, cardiac death, myocardial infarction, definite or probable stent thrombosis, and stroke between the very short term DAPT and standard DAPT groups in patients with diabetes (0.82, 0.64–1.04, *p* = 0.11; 0.77, 0.56–1.06, *p* = 0.10; 0.93, 0.75–1.17, *p* = 0.55; 0.92, 0.58–1.47, *p* = 0.74; and 0.97, 0.63–1.50, *p* = 0.89, respectively) (Figure 4). Similar outcomes above are also observed in patients with non-diabetes (Figure 4). The significant interaction of outcomes above was not observed between diabetes mellitus and non-diabetes mellitus subgroups. Meanwhile, no significant heterogeneity was tested by the *I*^2^, and no significant publication bias was found by the Begg's and Egger's test.

### Subgroup Analysis

#### Subgroup Analysis of Diabetes Management Strategy

For all patients, there was no significant difference in the incidence of major bleeding between insulin-dependent and non-insulin-dependent diabetes mellitus subgroups (1.32, 0.95–1.82, *p* = 0.1). However, the ischemic outcomes of all-cause death, cardiac death, and ischemic stroke were more likely to occur in patients with insulin-dependent diabetes mellitus (1.72, 1.32–2.24, *p* <0.0001; 1.63, 1.14–2.34, *p* = 0.08; 1.87, 1.05–3.31, *p* = 0.03, respectively). In addition, the outcome of bleeding and ischemia is not obvious in patients with or without insulin-dependent diabetes mellitus between very short term and long-term DAPTs (Supplementary Table 5).

#### Subgroup Analysis Based on Clinical Manifestation

Patients with acute coronary syndrome can benefit from the very term short DAPT at the outcome of major bleeding (0.56, 0.34–0.90, *p* = 0.02). Meantime, the same results were found in patients with non-diabetes (0.48, 0.25–0.93; *p* = 0.03). However, there was no significant difference in patients with diabetes. In addition, the very short term DAPT can reduce the incidence of the NACE in diabetic patients with any coronary artery disease (0.69, 0.55–0.86, *p* = 0.0009) (Supplementary Table 6).

#### Subgroup Analysis of P2Y_12_ or Aspirin Monotherapy After Very Short Term Dual Antiplatelet Therapy

Compared with aspirin monotherapy, P2Y_12_ monotherapy after very short term DAPT can significantly reduce the incidence of the NACE and major bleeding (0.76, 0.65–0.89, *p* = 0.0006 and 0.79, 0.66–0.94, *p* = 0.007) in all patients included. The same was true of the incidence of the NACE in patients with diabetes. However, there is no significant difference in other outcomes between the two subgroups regardless of diabetic status (Supplementary Table 7).

#### Subgroup Analysis of Different P2Y_12_ Antagonists in the Very Short Term Dual Antiplatelet Therapy

Both ticagrelor and clopidogrel can reduce the incidence of the NACE in the very short term DAPT (*p* = 0.02 and *p* = 0.04, respectively). However, compared with clopidogrel, ticagrelor can significantly reduce the incidence of the NACE in patients with diabetes (0.64, 0.50–0.82, *p* = 0.0005) (Supplementary Table 8).

## Discussion

In this meta-analysis of randomized controlled trials, the very short term DAPT is associated with a significant reduction in the incidence of the NACE and MACCE compared with standard DAPT in patients with diabetes. P2Y_12_ monotherapy after very short term DAPT can significantly reduce the incidence of the NACE, especially in patients with diabetes. Ticagrelor rather than clopidogrel combined with aspirin as the DAPT strategy may be suitable for patients with diabetes. Meanwhile, the very short term DAPT can also decrease the incidence of the major or minor bleeding outcome no matter what the diabetes mellitus status. Patients with acute coronary syndrome can benefit from the very short term DAPT at the major bleeding outcome. However, shortening the duration of DAPT had no significant effect on the incidence of all-cause death, cardiac death, major bleeding, myocardial infarction, definite or probable stent thrombosis, and stroke in both diabetic and non-diabetic patients.

The DES reduced the risk of clinical outcomes after PCI compared with bare metal stent (27). However, since the first-generation DES increased the risk of late and very late thrombosis (9), the concept of prolonging DAPT was proposed for a period of time. As the stent technology became more advanced, the second-generation DES has been shown to significantly reduce stent thrombosis regardless of DAPT duration (8, 28)_._ Moreover, prolonging the duration of DAPT increases the risk of bleeding events and medical costs. The AHA and ESC guidelines currently focus on the update of DAPT duration, and which suggests that a variety of treatment strategies for DAPT after PCI should depend on the type of coronary artery disease and the risk of bleeding. However, 1–3-month DAPT is only recommended for patients with stable coronary artery disease and without a high risk of bleeding no matter the status of diabetes (13, 14). In addition, the 2019 ESC guideline on diabetes and cardiovascular disease suggests that diabetes patients undergoing PCI should receive DAPT for at least 1 year, and patients with tolerable bleeding should be treated with DAPT for 3 years (1). Diabetes mellitus was identified to be a predictor of thrombotic events and was more prone to revascularization (10, 29). Meanwhile, it was considered a dependent variable of the DAPT score, which may need to prolong the duration of DAPT (30). However, a previous meta-analysis reported that the standard DAPT was associated with bleeding complications regardless of diabetic state (12). In recent years, the safety and effectiveness of de-escalation strategy of DAPT have been demonstrated by many randomized controlled trials (17, 19, 21, 22, 31, 32). Although there are no studies devoted to DAPT in patients with diabetes, a number of pre-specified subgroup analysis from the randomized studies have been published, which shows some contrasting results (15, 16). Furthermore, recent studies have shown that the very short DAPT did not increase ischemic events in patients with diabetes (15, 16). Nevertheless, it can reduce the NACE after second-generation DES implantation (18, 21, 33). In view of the above reasons, the optimal duration of DAPT in diabetic patients after PCI is still controversial. Therefore, it is very important to explore the effectiveness and safety of the very short term DAPT.

This meta-analysis showed that the very short term DAPT significantly reduced the incidence of the NACE in patients with diabetes. However, previous meta-analysis (DAPT ≤ 6 vs. 12 months) did not conclude similar results (12, 34). There are several reasons for this difference. Firstly, this study further reduced the duration of DAPT to 3 months, and the stents were limited to second-generation DES. Furthermore, this result was largely influenced by the TWILIGHT study, that is, through its large sample size and significant results. Although the result was based on low heterogeneity and negative publication bias, the specificity of the TWILIGHT trial population (the increased risk of bleeding and ischemia) needs to be considered and validated in a wider population. For the non-diabetes subgroup outcome of the NACE, the Egger's test showed publication bias, which needs more trials to confirm the results. In the subgroup of antiplatelet therapy after very short term DAPT, P2Y_12_ monotherapy can reduce the incidence of the NACE, especially in patients with diabetes. Compared with aspirin, P2Y_12_ had stronger antiplatelet function and can balance the ischemia and bleeding after transient DAPT (35). In addition, ticagrelor combined with aspirin may be better than clopidogrel combined with aspirin in patients with diabetes. Ticagrelor is a reversible binding oral direct-acting P2Y_12_ receptor antagonist, which has rapid onset and stronger inhibitory effect on platelet aggregation than clopidogrel. It may be more suitable for diabetic patients with high ischemia. These conclusions also are supported by the PLATO and THEMIS-PCI trials (36, 37).

For bleeding outcomes, the very short term DAPT significantly reduced the incidence of major or minor bleeding in both populations, but no such significant effect was observed in major bleeding outcome. However, the results of major bleeding still need to be interpreted under the premise of high heterogeneity. Despite that the sensitivity analysis did not change the statistical results, the different definitions of major bleeding included in the study and the strategy of P2Y_12_ inhibitor monotherapy should be considered when interpreting the results. In addition, the results of the GLOBAL LEADERS trial also need to be analyzed without meeting the expectation. The very short term DAPT can reduce the outcome of major or minor bleeding, with uniform definition of BARC 2,3,5 in patients with or without diabetes following second-generation DES. There is no heterogeneity in this outcome. However, since only two trials were included in the analysis, more evidence is needed to support the results. In the subgroup analysis of clinical manifestation, patients with acute coronary syndrome had a lower risk of major bleeding under the very short term DAPT. However, compared with patients with non-diabetes, patients with diabetes did not show the same effect. Moreover, current guidelines recommend that patients with acute coronary syndrome receive DAPT for at least 12 months, which need to be carefully evaluated based on the result of this analysis, especially in a population without a high ischemia risk.

In terms of ischemic outcomes, no difference was observed regardless of diabetic status, which is consistent with most previous analyses (12, 38). Nevertheless, a systematic review and meta-analysis by Sharma et al. (34) indicated that the short term DAPT (1–6 months) can increase the risk of stent thrombosis. This difference can be interpreted by the use of more first-generation stents and different monotherapy strategies after DAPT. In addition, compared with non-insulin-dependent diabetic patients, insulin-dependent diabetic patients were associated with a higher risk of ischemic outcome. However, the duration of antiplatelet therapy did not affect the outcome of bleeding or ischemia, regardless of diabetes management strategy.

The results of this meta-analysis need to be interpreted carefully. Firstly, all the included studies were in the subgroup of randomized controlled trial, and only part of them was a pre-specified subgroup or stratified by diabetes. Secondly, this meta-analysis included acute coronary syndrome and stable coronary artery disease patients with or without diabetes; majority of studies came from multiple countries or centers, including the high-risk population of bleeding and ischemia, which was representative. However, the data of insulin-dependent and non-insulin-dependent diabetes were only from two randomized controlled trials, and more data are needed to confirm the results. Finally, there were no absolutely stable coronary artery disease patients in any trial. Therefore, the results of this analysis were not suitable for these patients. There were no other types of P2Y_12_ except ticagrelor and clopidogrel in the subgroup analysis. Although most of the studies included were open label, all the outcomes were moderate in the quality assessment of GRADE evidence, which improves the reliability of the results to a certain extent. However, it is expected that randomized controlled trials focused on diabetes mellitus will be performed to confirm this meta-analysis.

### Limitations

This meta-analysis also has some limitations. First of all, one of the major issues is that there were few studies dedicated to diabetes. However, all diabetic patients who received second-generation DES were treated in the subgroup of randomized controlled trials. This may reduce the reliability of the results to some extent. In addition, the subgroup studies often have incomplete outcome reported, which has an impact on data collection. Many outcomes were not included in all eight studies, which could reduce the stability of outcomes. Thirdly, although patients undergoing second-generation DES were only included in this meta-analysis, there are still many differences in the second-generation DES in the world, which may lead to confusion of interpreting results. Last but not least, the inconsistency of study design, such as different population, the duration of DAPT, and drug used, may affect the interpretation of the results. Although subgroup analysis was performed to explore the differences of its impact on the results, this can only be used as reference, not as evidence.

### Conclusion

The current guidelines emphasize that the duration of DAPT should be based on the risk assessment of bleeding and ischemia. The meta-analysis reported the application of very short term DAPT in diabetic patients implanted with second-generation DES, which suggests that the very short term DAPT can significantly improve the outcomes of the NACE and MACCE in patients with diabetes. The regimen can reduce the risk of major or minor bleeding without increasing the risk of ischemia, regardless of the state of diabetes. For patients with diabetes, ticagrelor combined with aspirin as a strategy of DAPT, and P2Y_12_ instead of aspirin monotherapy after very short term DAPT may be desirable choices.

## Data Availability Statement

The original contributions presented in the study are included in the article/Supplementary Material, further inquiries can be directed to the corresponding author/s.

## Author Contributions

X-YL and YL contributed to the conception and method of the work. X-YL, YL, and W-JZ acquired and extracted the data. X-YL and XQ analyzed the data with corresponding software. X-YL and Z-LW wrote, reviewed, and edited the manuscript. All authors read and approved the final manuscript.

## Conflict of Interest

The authors declare that the research was conducted in the absence of any commercial or financial relationships that could be construed as a potential conflict of interest.
