# Supplementary material for: Clinical Outcomes of Very Short Term Dual Antiplatelet Therapy in Patients With or Without Diabetes Undergoing Second-Generation Drug-Eluting Stents: A Systematic Review and Meta-Analysis of Randomized Clinical Trials
Source: Front Cardiovasc Med. 2021 Jul 1;8:655718. doi: 10.3389/fcvm.2021.655718 (PMC8281288; doi:10.3389/fcvm.2021.655718)
Supplement: Supplementary file 1 [file Data_Sheet_1.pdf]

# SUPPLEMENTARY DATA

## Clinical outcomes of very short term dual antiplatelet therapy in patients with or without diabetes undergoing second-generation drug-eluting stents: a systematic review and meta-analysis of randomized clinical trials

## List of Supporting Information Content

**Supplementary Table 1.** Outcome definitions of randomized controlled trials included.

**Supplementary Table 2.** Inclusion, exclusion criteria and primary outcomes of studies included.

**Supplementary Table 3.** Clinical, angiographic and procedural characteristics of patients with or without diabetes.

**Supplementary Table 4.** Summary of GRADE evidence quality evaluation.

**Supplementary Table 5.** Subgroup analysis of diabetes management strategy.

**Supplementary Table 6.** Subgroup analysis based on clinical manifestation.

**Supplementary Table 7.** Subgroup analysis of $P2Y_{12}$ or aspirin monotherapy after very short term DAPT.

**Supplementary Table 8.** Subgroup analysis of different $P2Y_{12}$ antagonists in the very short term DAPT.

**Supplementary Figure 1.** Quality assessment of each randomized controlled trial

included.

**Supplementary Figure 2.** The funnel plot of NACE outcome.

**Supplementary Figure 3.** Sensitivity analysis for outcome of major bleeding in patients with diabetes.

**Supplementary Table 1.** Outcome definitions of the randomized controlled trials included.

| Study | NACE | MACCE | cardiac death | MI | Stroke | ST | Bleeding |
|---|---|---|---|---|---|---|---|
| TICO | MACCE plus major bleeding as defined by TIMI. | Composite outcome of all-cause death, MI, ST, stroke, and TVR. | death due to MI, cardiac perforation or pericardial tamponade, an arrhythmia or conduction abnormality, stroke within 30 days of the procedure or related to the procedure, death due to a procedural complication. | Third universal definition | An acute cerebrovascular event resulting in death or neurological deficit＞24 hours or the presence of acute infarction demonstrated by imaging studies. | ARC criteria | TIMI criteria. |
| TWILIGHT | - | - | Any death due to proximate cardiac cause, unwitnessed death and death of unknown cause, all procedure-related deaths including those related to concomitant treatment. | Third universal definition | acute symptomatic episode of neurological dysfunction, more than 24 hours in duration in the absence of therapeutic intervention or death, due to cerebral, spinal or retinal tissue injury as evidenced by neuroimaging or lumbar puncture. | ARC criteria | BARC criteria. |

| | | | | | | | |
|---|---|---|---|---|---|---|---|
| STOPDAPT -2 | cardiovascular death, MI, definite ST stroke, or TIMI major or minor bleeding. | cardiovascular end point (a composite of cardiovascular death, MI, definite ST, or stroke. | - | ARC criteria. | | ARC criteria | TIIMI criteria. |
| SMART-CHOICE | - | - | All deaths were considered cardiac unless a definite non-cardiac cause could be established. | Elevated cardiac enzyme levels above the URL with ischemic symptoms or ECG findings indicative of ischemia. | Non- convulsive focal or global neurologic deficit of abrupt onset lasting for more than 24 hours or leading to death, which was caused by ischemia or hemorrhage within the brain. | ARC criteria | BARC criteria. |
| REDUCE | MACCE plus BARC 2 to 5 bleeding. | - | - | Third universal definition. | - | ARC criteria . | BARC criteria. |

| | | | | | | | |
|---|---|---|---|---|---|---|---|
| GLOBAL-LEADERS | - | - | - | Third universal definition. | - | | BARC criteria. |
| OPTIMIZE | - | - | Any death unless a definite non-cardiovascular cause could be established. | Per-procedural: rise in cardiac enzyme within 48hs after PCI or >5 times ULN, with new Q wave/LBBB after CABG. | Acute neurological event with duration ⩾24hs with confirmation by either CT or MRI or pathological confirmation. | ARC criteria. | REPLACE criteria. |
| RESET | composite of cardiovascular death, MI, ST, TVR, or bleeding. | - | Any death unless a definite non-cardiovascular cause could be established. | Typical symptoms, with EKG changes and a rise in cardiac enzyme*, unrelated to an interventional procedure. | Sudden onset of vertigo, numbness, aphasia, or dysarthria resulting from vascular lesions of the brain, including hemorrhage, embolism, thrombosis, or rupturing aneurysm. | ARC criteria. | TIIMI criteria. |

Abbreviations: ST= stent thrombosis , ARC=Academic Research Consortium; BARC=Bleeding Academic Research Consortium; CABG=coronary artery bypass grafting; CPK-MB= creatine phosphokinase myocardial band; CT=computer tomography; EKG=electrocardiography; LBB=left bundle branch block; MRI= magnetic resonance imaging; PCI=percutaneous coronary intervention; REPLACE=Randomized Evaluation of PCI Linking Angiomax to Reduced Clinical Events; TIMI=thrombolysis in myocardial infarction; TVR=target vessel revascularization; ULN=upper limit of normal.

*levels CPK-MB >3 times the ULN range or troponin T/I > than the 99th percentile of the ULN

**Supplementary Table 2.** Inclusion, exclusion criteria and primary outcomes of studies included.

| Study | Major inclusion criteria | Major exclusion criteria | Primary outcomes | Primary Outcomes Results |
|---|---|---|---|---|
| TICO | ACS implanted bioresorbable polymer sirolimus-eluting stent. | Increased risk of bleeding, need for oral anticoagulation therapy, currently treated with strong CYP3A4 inhibitors. | composite of major bleeding (TIMI) and adverse cardiac and cerebrovascular events (death, MI, ST, stroke, and TVR). | superiority demonstrated. |
| TWILIGHT | High-risk patients who have undergone successful PCI with at least 1 locally approved drug-eluting stent discharged on DAPT with aspirin and ticagrelor for at least 3 month. | Need for oral anticoagulants; History of intracranial hemorrhage. | Bleeding Academic Research Consortium (BARC) 2, 3 or 5 bleeding. | Non-inferiority demonstrated. |
| STOPDAPT-2 | Patients who have undergone PCI with the everolimus-eluting cobalt-chromium stent and have not experienced major complications during hospital stay for treatment. | requiring oral anticoagulants, history of intracranial hemorrhage, experienced major complications during hospital stay post-PCI. | composite of cardiovascular death, MI, ischemic or hemorrhagic stroke, definite ST, or major or minor bleeding. | Non-inferiority and superiority demonstrated. |
| SMART-CHOICE | stable ischemic or ACS with more than 50% diameter stenosis and a visually estimated diameter of ≥2.25. mm and ≤4.25 mm | cardiogenic shock, active pathologic bleeding, history of DES implantation within 12 months. | composite of all-cause death, MI, or stroke. | Non-inferiority demonstrated. |

| | | | | |
|---|---|---|---|---|
| REDUCE | ACS patients successfully treated with COMBO stent. | cardiogenic shock, recent major bleeding complications or contraindication to DAPT. | composite of all-cause mortality, MI, ST, stroke, TVR and bleeding. | Non-inferiority demonstrated. |
| GLOBAL-LEADERS | any clinical indication for percutaneous coronary intervention, with more than 50% diameter stenosis. | fibrinolytic therapy within 24 hours of PCI, scheduled elective surgery within 12 months, previous stent thrombosis, stroke within last 30 days. | all-cause death or new Q-wave MI. | superiority not demonstrated. |
| OPTIMIZE | Stable angina or low risk unstable angina with at least 1 lesion in native coronary vessel ⩾2.5 mm in diameter. | STEMI, scheduled elective surgery within 12 months, in stent restenosis of DES, BMS in non-target vessel in the last 6 months. | Death, MI, CVA(stroke) or major bleeding. | Non-inferiority demonstrated. |
| RESET | Stable, unstable angina or acute MI with more than 50% diameter stenosis in a coronary artery. | Cardiogenic shock, STEMI within 48 hours, LVEF<40%, previous stent thrombosis, CTO. | Cardiac death, MI, ST, TVR or major bleeding. | Non-inferiority demonstrated. |

**Abbreviations:** ACS=acute coronary syndrome; CYP3A4=cytochrome P450 3A4; DAPT=dual antiplatelet therapy; TIMI= thrombolysis in myocardial infarction; MI=myocardial infarction; ST=stent thrombosis; TVR=target vascular revascularization; STEMI=ST-segment elevated myocardial infarction; PCI=percutaneous coronary intervention; DES=drug eluting stents; CTO=chronic total occlusion; BARC=Bleeding Academic Research Consortium.

**Supplementary Table 3.** Clinical, angiographic and procedural characteristics of patients with or without diabetes.

| Study | TWILIGHT | | GLOBEL-LEADERS | | *P* |
|---|---|---|---|---|---|
| | Diabetes (N=2620) | Non-diabetes (N=4499) | Diabetes (N=4038) | Non-diabetes (N=11919) | |
| Age, years mean ± SD | 64.8±10.1 | 65.4±10.1 | 66.33±9.48 | 63.93±10.5 | ＜0.0001 |
| Female | 23.6 | 24.0 | 26.4 | 22.18 | ＜0.0001 |
| BMI, kg/m$^2$, mean ± SD | 29.8±6.0 | 27.9±5.2 | 29.66±5.01 | 27.69±4.33 | ＜0.0001 |
| chronic kidney disease | 21.1 | 13.6 | 20.81 | 11.25 | ＜0.0001 |
| Anemia | 25.6 | 15 | 21.31 | 10.76 | ＜0.0001 |
| Current Smoker | 18.1 | 23,9 | 19.81 | 28.22 | ＜0.0001 |
| Hypercholesterolemia | 66.6 | 56.9 | 78.38 | 66.65 | ＜0.0001 |
| Hypertension | 81.5 | 67.1 | 86.69 | 69.16 | ＜0.0001 |
| Peripheral arterial disease | 8.4 | 6.0 | 10.04 | 5.11 | ＜0.0001 |
| Previous MI | 29.3 | 28.3 | 27.0 | 22.06 | ＜0.0001 |
| Previous PCI | 45.5 | 40.1 | 40.22 | 30.2 | ＜0.0001 |
| Previous CABG | 12.6 | 8.4 | 9.25 | 4.97 | ＜0.0001 |
| Previous major bleeding | 0.9 | 0.9 | 0.6 | 0.62 | 0.79 |
| Clinical presentation | | | | | 0.0036 |
| Stable coronary artery disease | 1006(38.4) | 509(33.3) | 2434(60.28) | 6041(50.68) | |
| Acute coronary syndrome | 1614(61.6) | 810(66.7) | 1604(39.72) | 5878(49.32) | |
| Target Vessel | | | | | |
| Left Main | 4.7 | 5.1 | 2.92 | 2.64 | 0.25 |
| LAD | 55.5 | 56.7 | 48.98 | 51.47 | 0.07 |
| LCX | 33.9 | 31.3 | 33.47 | 30.99 | ＜0.0001 |
| RCA | 35 | 35.2 | 36.81 | 37.84 | 0.16 |
| Number of lesion treated, mean ± SD | 1.5±0.8 | — | 1.46±0.75 | 1.43±0.74 | 0.05 |

| | | | | | |
|---|---|---|---|---|---|
| Total stent length(mm), mean ± SD | 39.5 ±24.0 | 40.1 ±24.4 | 36.21 ±25.54 | 35.24 ±25.14 | 0.01 |
| Minimum stent diameter(mm), mean ± SD | 2.8 ±0.5 | 2.9 ±0.5 | — | — | ＜0.0001 |
| Stent number per patient | — | — | 1.75 ±1.09 | 1.71 ±1.08 | ＜0.0001 |
| Bifurcation PCI | 11.6 | 12.5 | 14.74 | 16.1 | 0.02 |
| Multi-vessel CAD/PCI | 68.6 | 59.3 | 23.23 | 22.36 | 0.11 |
| Average stent diameter per patient (mm), mean ± SD | — | — | 2.96 ±0.43 | 3.01 ±0.43 | ＜0.0001 |

**Abbreviations:** LAD= left anterior descending, LCX= left circumflex artery, RCA= Right coronary artery. PCI=percutaneous coronary intervention. MI=myocardial infarction, CABG= Coronary Artery Bypass Grafting. CAD=coronary artery disease. Values shown are % (n) otherwise indicated.

**Supplementary Table 4.** Summary of GRADE evidence quality evaluation.

**Patient or population:** patients with diabetes following percutaneous coronary intervention.
**Settings:**
**Intervention:** De-escalation dual antiplatelet therapy.
**Comparison:** standard dual antiplatelet therapy.

| Outcomes | Illustrative comparative risks* (95% CI) | | Relative effect (95% CI) | No of Participants (studies) | Quality of the evidence (GRADE) | Comments |
|---|---|---|---|---|---|---|
| | Assumed risk | Corresponding risk | | | | |
| | **Standard dual antiplatelet therapy** | **De-escalation dual antiplatelet therapy** | | | | |
| **net adverse clinical events** Follow-up: median 12 months | **Study population** | | **RR 0.72** (0.6 to 0.88) | 6311 (6 studies) | ⊕⊕⊕⊖ **moderate**[1] | |
| | **74 per 1000** | **53 per 1000** (44 to 65) | | | | |
| | **Low** | | | | | |
| | **44 per 1000** | **31 per 1000** (26 to 38) | | | | |
| | **High** | | | | | |
| | **104 per 1000** | **75 per 1000** (62 to 92) | | | | |
| **major bleeding** Follow-up: median 12 months | **Study population** | | **RR 0.69** (0.35 to 1.36) | 8596 (4 studies) | ⊕⊕⊕⊖ **moderate**[1] | |
| | **25 per 1000** | **17 per 1000** (9 to 34) | | | | |
| | **Low** | | | | | |

| | | | | | |
|---|---|---|---|---|---|
| | **High** | | | | |
| | **43 per 1000** | **30 per 1000** (15 to 58) | | | |
| **major or minor bleeding** Follow-up: median 12 months | **Study population** | | **RR 0.69** (0.52 to 0.93) | 3742 (2 studies) | ⊕⊕⊕⊖ **moderate**[1] |
| | **55 per 1000** | **38 per 1000** (29 to 51) | | | |
| | **Low** | | | | |
| | **29 per 1000** | **20 per 1000** (15 to 27) | | | |
| | **High** | | | | |
| | **66 per 1000** | **46 per 1000** (34 to 61) | | | |
| **major cardiac or cerebrovascular events** Follow-up: median 12 months | **Study population** | | **RR 0.87** (0.77 to 0.98) | 8616 (4 studies) | ⊕⊕⊕⊖ **moderate**[1] |
| | **114 per 1000** | **99 per 1000** (88 to 112) | | | |
| | **Low** | | | | |
| | **36 per 1000** | **31 per 1000** (28 to 35) | | | |
| | **High** | | | | |
| | **186 per 1000** | **162 per 1000** (143 to 182) | | | |
| **all-cause death** Follow-up: median 12 months | **Study population** | | **RR 0.82** (0.64 to | 7707 (3 studies) | ⊕⊕⊕⊖ **moderate**[1] |
| | **37 per 1000** | **30 per 1000** | | | |

| | | | | | |
|---|---|---|---|---|---|
| | | (23 to 38) | 1.04) | | |
| | **Low** | | | | |
| | **20 per 1000** | **16 per 1000**<br>(13 to 21) | | | |
| | **High** | | | | |
| | **48 per 1000** | **39 per 1000**<br>(31 to 50) | | | |
| **cardiac death**<br>Follow-up: median 12 months | **Study population** | | **RR 0.77**<br>(0.56 to 1.06) | 7999<br>(4 studies) | ⊕⊕⊕⊝<br>**moderate**[1] |
| | **22 per 1000** | **17 per 1000**<br>(12 to 23) | | | |
| | **Low** | | | | |
| | **7 per 1000** | **5 per 1000**<br>(4 to 7) | | | |
| | **High** | | | | |
| | **27 per 1000** | **21 per 1000**<br>(15 to 29) | | | |
| **myocardial infarction**<br>Follow-up: median 12 months | **Study population** | | **RR 0.93**<br>(0.75 to 1.17) | 7999<br>(4 studies) | ⊕⊕⊕⊝<br>**moderate**[1] |
| | **39 per 1000** | **36 per 1000**<br>(29 to 45) | | | |
| | **Low** | | | | |
| | **7 per 1000** | **6 per 1000**<br>(5 to 8) | | | |
| | **High** | | | | |

| | | | | | |
|---|---|---|---|---|---|
| | **41 per 1000** | **38 per 1000**<br>(31 to 48) | | | |
| **stent thrombosis**<br>Follow-up: median 12 months | **Study population** | | **RR 0.92**<br>(0.58 to 1.47) | 9158<br>(5 studies) | ⊕⊕⊕⊝<br>**moderate**[1] |
| | **8 per 1000** | **7 per 1000**<br>(5 to 12) | | | |
| | **Low** | | | | |
| | **2 per 1000** | **2 per 1000**<br>(1 to 2) | | | |
| | **High** | | | | |
| | **11 per 1000** | **10 per 1000**<br>(6 to 16) | | | |
| **stroke**<br>Follow-up: median 12 months | **Study population** | | **RR 0.97**<br>(0.63 to 1.5) | 7707<br>(3 studies) | ⊕⊕⊕⊝<br>**moderate**[1] |
| | **11 per 1000** | **10 per 1000**<br>(7 to 16) | | | |
| | **Low** | | | | |
| | **4 per 1000** | **3 per 1000**<br>(2 to 5) | | | |
| | **High** | | | | |
| | **17 per 1000** | **16 per 1000**<br>(10 to 25) | | | |

*The basis for the **assumed risk** (e.g. the median control group risk across studies) is provided in footnotes. The **corresponding risk** (and its 95% confidence interval) is based on the assumed risk in the comparison group and the **relative effect** of the intervention (and its 95% CI).

**CI:** Confidence interval; **RR:** Risk ratio;

GRADE Working Group grades of evidence

**High quality:** Further research is very unlikely to change our confidence in the estimate of effect.

**Moderate quality:** Further research is likely to have an important impact on our confidence in the estimate of effect and may change the estimate.

**Low quality:** Further research is very likely to have an important impact on our confidence in the estimate of effect and is likely to change the estimate.

**Very low quality:** We are very uncertain about the estimate.

[1] Most of included trials were open label.

**Supplementary Table 5.** Subgroup analysis of diabetes management strategy.

| | insulin dependent | | | | non-insulin dependent | | | | | |
|---|---|---|---|---|---|---|---|---|---|---|
| | Total (n=1932) | 1-3 months (n=940) | 12 months (n=991) | $P^a$ | Total (n=4710） | 1-3 months (n=2408) | 12 months (n=2282) | $P^b$ | RR [95% CI] | $P^c$ |
| Bleeding outcome | | | | | | | | | | |
| major bleeding | 54 (2.80%) | 28 (2.98%) | 26 (2.62%) | 0.94 | 99 (2.10%) | 38 (1.86%) | 61 (2.67%) | 0.26 | 1.32 [0.95, 1.83] | 0.1 |
| Ischemic outcomes | | | | | | | | | | |
| all-cause death | 90 (4.66%) | 37 (3.94%) | 53 (5.35%) | 0.12 | 124 (2.63%) | 30 (1.46%) | 32 (1.40%) | 0.31 | 1.72 [1.32, 2.24] | ＜0.0001 |
| cardiac death | 49 (2.54%) | 37 (3.94%) | 50 (2.62%) | 0.14 | 72 (1.53%) | 15 (0.73%) | 19 (0.83%) | 0.42 | 1.63 [1.14, 2.34] | 0.008 |
| myocardial infarction | 95 (4.92%) | 51 (5.43%) | 44 (2.62%) | 0.57 | 168 (3.57%) | 79 (3.86%) | 89 (3.90%) | 0.26 | 1.43 [0.94, 2.18] | 0.1 |
| ischemic stroke | 34 (1.76%) | 17 (1.81%) | 14 (2.62%) | 0.52 | 45 (0.96%) | 19 (0.93%) | 19 (0.83%) | 0.85 | 1.87 [1.05, 3.31] | 0.03 |
| definite or probable ST | 14 (0.72%) | 6 (0.64%) | 8 (2.62%) | 0.69 | 45 (0.96%) | 23 (1.12%) | 22 (0.96%) | 0.98 | 0.75 [0.41, 1.36] | 0.34 |

The data was summarized from trials of GLOBAL-LEADERS and TWILIGHT. [a] *P* value for insulin dependent diabetes patients according to duration of DAPT, [b] *P* value for non-insulin dependent diabetes patients according to duration of DAPT, [c] *P* value for outcomes dependent on management of diabetes. ST=stent thrombosis.

**Supplementary Table 6.** Subgroup analysis based on clinical manifestation.

| | | 1-3 months | 12 months | RR [95% CI] | *P* |
|---|---|---|---|---|---|
| Total | | | | | |
| NACE | ACS | 119/2255 | 150/2262 | 0.81 [0.55, 1.19] | 0.28 |
| | any CAD | 333/7777 | 389/7787 | 0.86 [0.73, 1.01] | 0.06 |
| major bleeding | ACS | 25/1527 | 45/1529 | 0.56 [0.34, 0.90] | 0.02 |
| | any CAD | 202/13092 | 244/13103 | 0.73 [0.42, 1.25] | 0.25 |
| MACCE | ACS | 35/1527 | 51/1529 | 0.69 [0.45, 1.05] | 0.08 |
| | any CAD | 1226/13021 | 1303/13046 | 0.94 [0.87, 1.01] | 0.1 |
| Diabetes mellitus | | | | | |
| NACE | ACS | 44/576 | 51/562 | 0.84 [0.57, 1.24] | 0.38 |
| | any CAD | 126/2604 | 181/2507 | 0.69 [0.55, 0.86] | 0.0009 |
| major bleeding | ACS | 12/418 | 18/417 | 0.67 [0.32, 1.36] | 0.27 |
| | any CAD | 67/3922 | 87/3839 | 0.71 [0.26, 2.00] | 0.52 |
| MACCE | ACS | 14/418 | 21/417 | 0.67 [0.34, 1.29] | 0.23 |
| | any CAD | 420/3938 | 464/3843 | 0.88 [0.78, 1.00] | 0.04 |
| Non- diabetes mellitus | | | | | |
| NACE | ACS | 75/1679 | 99/1701 | 0.77 [0.52, 1.16] | 0.21 |
| | any CAD | 207/5173 | 208/5217 | 0.98 [0.77, 1.24] | 0.87 |
| major bleeding | ACS | 13/1109 | 27/1112 | 0.48 [0.25, 0.93] | 0.03 |
| | any CAD | 202/13092 | 244/13103 | 0.73 [0.42, 1.25] | 0.25 |
| MACCE | ACS | 21/1109 | 30/1112 | 0.70 [0.40, 1.22] | 0.21 |
| | any CAD | 806/9083 | 839/9203 | 1.03 [0.85, 1.24] | 0.79 |

**Abbreviations:** ACS=acute coronary syndrome, CAD=coronary artery disease, NACE=net adverse clinical event, MACCE= major cardiac or cerebrovascular events.

**Supplementary Table 7.** Subgroup analysis of $P2Y_{12}$ or aspirin monotherapy after very short term DAPT.

| Total | | 1-3 months | 12 months | RR [95% CI] | *P* |
|---|---|---|---|---|---|
| NACE | $P2Y_{12}$ | 257/6582 | 340/6602 | 0.76 [0.65, 0.89] | 0.0006 |
| | aspirin | 195/3450 | 199/3447 | 0.98 [0.81, 1.19] | 0.83 |
| all-cause death | $P2Y_{12}$ | 258/11498 | 297/11471 | 0.87 [0.73, 1.02] | 0.09 |
| | aspirin | 43/1563 | 45/1556 | 0.95 [0.63, 1.44] | 0.81 |
| cardiac death | $P2Y_{12}$ | 127/11498 | 147/11471 | 0.86 [0.68, 1.09] | 0.22 |
| | aspirin | 31/2622 | 36/2614 | 0.86 [0.53, 1.38 | 0.53 |
| MI | $P2Y_{12}$ | 343/11498 | 345/11471 | 0.99 [0.86, 1.15] | 0.91 |
| | aspirin | 51/2622 | 46/2614 | 1.11 [0.71, 1.64] | 0.62 |
| ST | $P2Y_{12}$ | 100/12998 | 102/12980 | 0.98 [0.74, 1.29] | 0.88 |
| | aspirin | 9/2622 | 7/2614 | 1.28 [0.48, 3.44] | 0.62 |
| stroke | $P2Y_{12}$ | 104/11498 | 111/11471 | 0.93 [0.72, 1.22] | 0.62 |
| | aspirin | 5/1553 | 5/1556 | 1.00 [0.29, 3.43] | 0.99 |
| major bleeding | $P2Y_{12}$ | 222/13056 | 283/13076 | 0.79 [0.66, 0,94] | 0.007 |
| | aspirin | 5/1563 | 6/1556 | 0.83 [0.25, 2.71] | 0.76 |
| Diabetes mellitus | | | | | |
| NACE | $P2Y_{12}$ | 114/2322 | 173/2292 | 0.65 [0.52, 0.82] | 0.0002 |
| | aspirin | 56/858 | 59/839 | 0.92 [0.65, 1.32] | 0.66 |
| all-cause death | $P2Y_{12}$ | 96/3357 | 120/3247 | 0.78 [0.60, 1.01] | 0.06 |
| | aspirin | 21/554 | 19/549 | 1.10 [0.60, 2.01] | 0.77 |
| cardiac death | $P2Y_{12}$ | 52/3357 | 70/3247 | 0.72 [0.50, 1.03] | 0.07 |
| | aspirin | 16/700 | 16/695 | 0.99 [0.50, 1.96] | 0.98 |
| MI | $P2Y_{12}$ | 130/3357 | 134/3247 | 0.91 [0.64, 1.30] | 0.61 |
| | aspirin | 17/700 | 19/695 | 0.90 [0.47, 1.70] | 0.74 |
| ST | $P2Y_{12}$ | 30/3942 | 32/3821 | 0.91 [0.55, 1.50] | 0.71 |

| | | | | | |
|---|---|---|---|---|---|
| | aspirin | 4/700 | 4/695 | 1.03 [0.27, 3.99] | 0.96 |
| stroke | $P2Y_{12}$ | 39/3357 | 38/3247 | 0.99 [0.63, 1.55] | 0.97 |
| | aspirin | 1/554 | 2/549 | 0.50 [0.05, 5.45] | 0.57 |
| major bleeding | $P2Y_{12}$ | 78/3786 | 105/3707 | 0.64 [0.31, 1.32] | 0.23 |
| | aspirin | 1/554 | 0/549 | 2.97 [0.12, 72.87] | 0.5 |
| Non- diabetes mellitus | | | | | |
| NACE | $P2Y_{12}$ | 143/4260 | 167/4310 | 0.77 [0.49, 1.22] | 0.27 |
| | aspirin | 139/2592 | 140/2608 | 1.00 [0.80, 1.26] | 0.99 |
| all-cause death | $P2Y_{12}$ | 162/8141 | 177/8224 | 0.93 [0.75, 1.14] | 0.47 |
| | aspirin | 22/1009 | 26/1007 | 0.84 [0.48, 1.48] | 0.55 |
| cardiac death | $P2Y_{12}$ | 75/8141 | 77/8224 | 0.91 [0.53, 1.55] | 0.72 |
| | aspirin | 15/1922 | 20/1919 | 0.76 [0.29, 1.48] | 0.42 |
| MI | $P2Y_{12}$ | 213/8141 | 211/8224 | 1.06 [0.80, 1.40] | 0.71 |
| | aspirin | 34/1922 | 27/1919 | 1.26 [0.76, 2.08] | 0.37 |
| Probable or definite ST | $P2Y_{12}$ | 70/9056 | 70/9159 | 0.99 [0.71,1.38] | 0.96 |
| | aspirin | 5/1922 | 3/1919 | 1.60 [0.36, 7.02] | 0.53 |
| stroke | $P2Y_{12}$ | 65/8141 | 73/8224 | 1.24 [0.41, 3.74] | 0.70 |
| | aspirin | 4/1009 | 3/1007 | 1.33 [0.30, 5.93] | 0.71 |
| major bleeding | $P2Y_{12}$ | 144/9270 | 178/9369 | 0.75 [0.52, 1.07] | 0.11 |
| | aspirin | 4/1009 | 6/1007 | 0.67 [0.19, 2.35] | 0.53 |

**Abbreviations:** NACE=net adverse clinical event, MI=myocardial infarction, ST= stent thrombosis.

**Supplementary Table 8.** Subgroup analysis of different $P2Y_{12}$ antagonists in the very short term DAPT.

| Total | | 1-3 months | 12 months | RR [95% CI] | *P* |
|---|---|---|---|---|---|
| NACE | Ticagrelor | 222/5081 | 285/5093 | 0.77 [0.62, 0.95] | 0.02 |
| | Clopidogrel | 35/1500 | 55/1509 | 0.64 [0.42, 0.97] | 0.04 |
| Probable or definite ST | Ticagrelor | 96/11498 | 101/11471 | 0.95 [0.72, 1.26] | 0.72 |
| | Clopidogrel | 4/1500 | 1/1509 | 0.96 [0.67, 1.38] | 0.21 |
| Diabetes mellitus | | | | | |
| NACE | Ticagrelor | 96/1727 | 148/1718 | 0.64 [0.50, 0.82] | 0.0005 |
| | Clopidogrel | 18/585 | 25/574 | 0.71 [0.39, 1.28] | 0.25 |
| Probable or definite ST | Ticagrelor | 29/3357 | 31/3247 | 0.91 [0.55, 1.51] | 0.71 |
| | Clopidogrel | 1/585 | 1/574 | 0.98 [0.06, 15.65] | 0.99 |
| Non- diabetes mellitus | | | | | |
| NACE | Ticagrelor | 126/3345 | 137/3375 | 0.85 [0.48, 1.51] | 0.59 |
| | Clopidogrel | 17/915 | 30/935 | 0.58 [0.32, 1.04] | 0.07 |
| Probable or definite ST | Ticagrelor | 67/8141 | 70/8824 | 0.97 [0.69, 1.35] | 0.85 |
| | Clopidogrel | 3/915 | 0/935 | 7.15 [0.37, 138.28] | 0.19 |

**Abbreviations:** NACE=net adverse clinical event, ST= stent thrombosis.

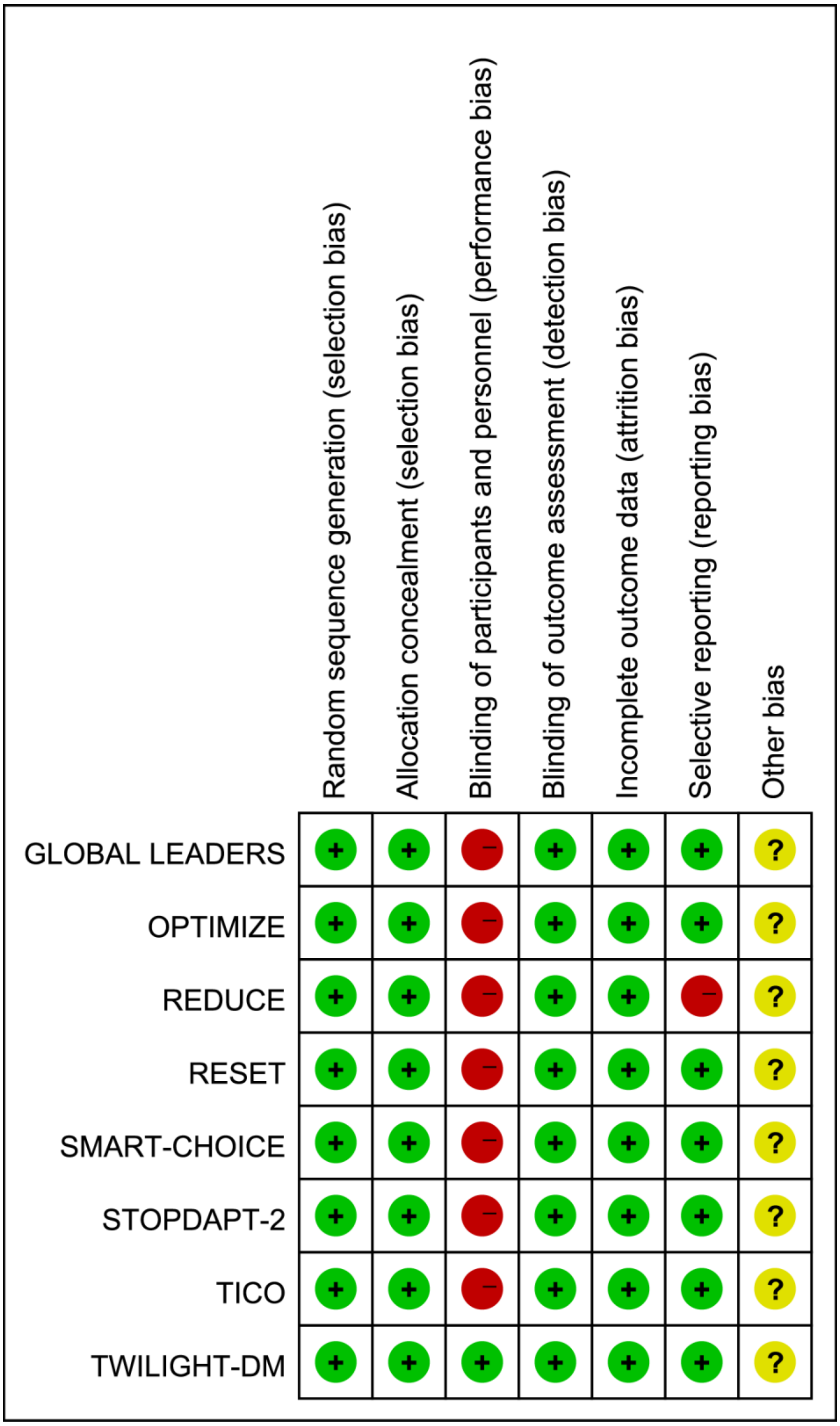


**Supplementary Figure 1.** Quality assessment of each randomized controlled trial included.

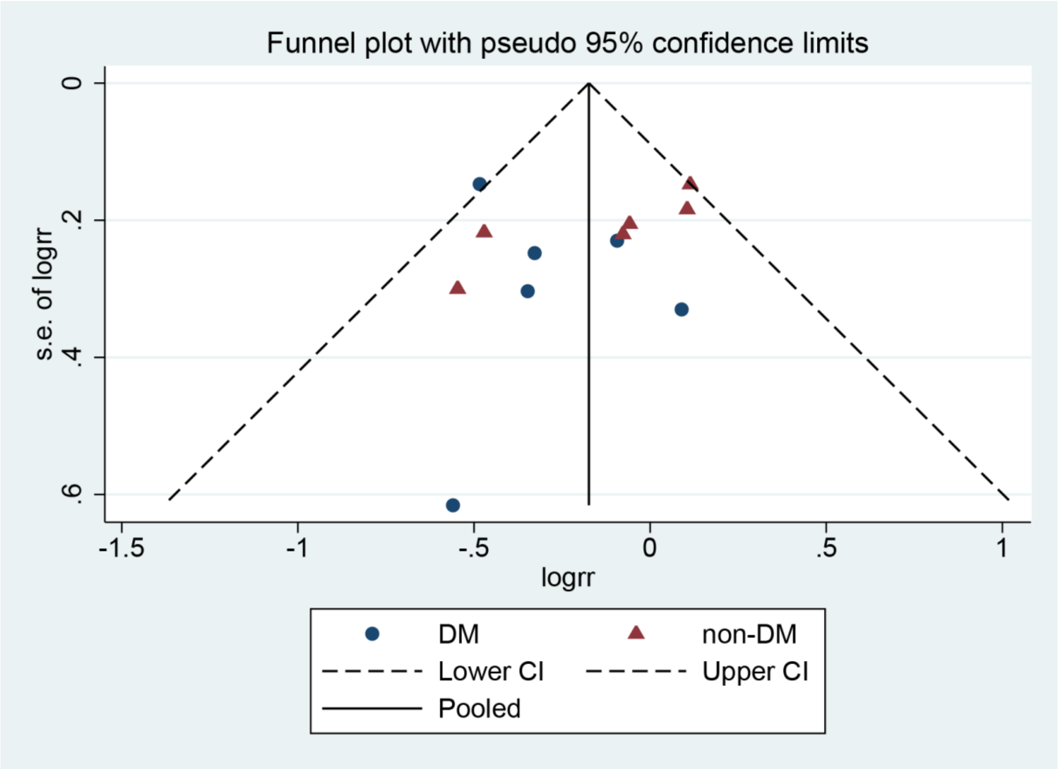


**Supplementary Figure 2.** The funnel plot of NACE outcome. (DM= diabetes mellitus, non-DM=non- diabetes mellitus.)

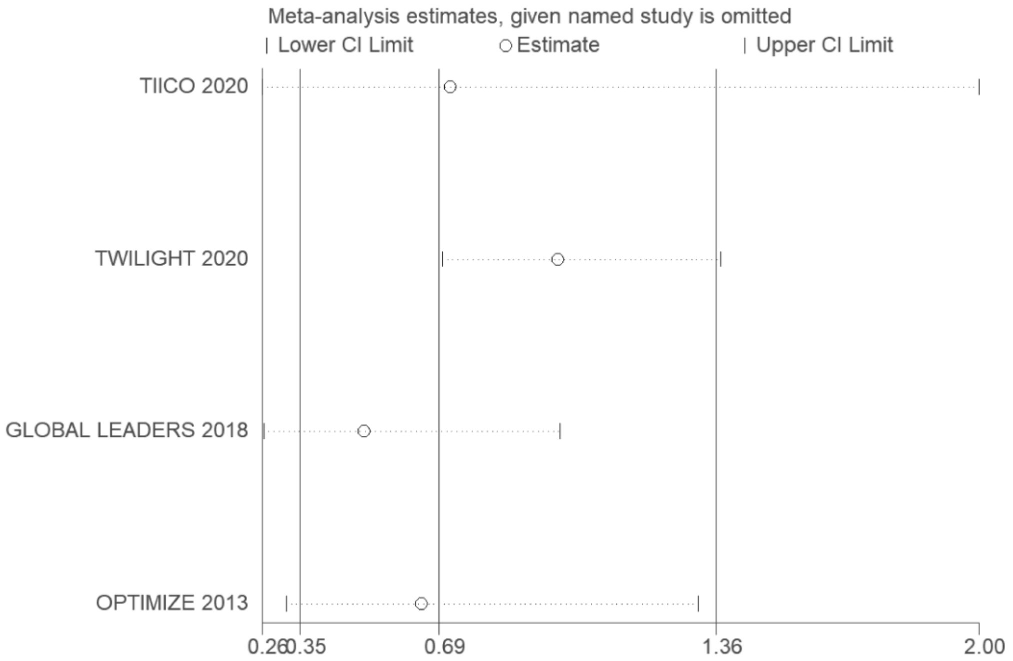


**Supplementary Figure 3.** Sensitivity analysis for outcome of major bleeding in patients with diabetes mellitus.
